# Investigating immersion and migration decisions for agent-based modelling: A cautionary tale

**DOI:** 10.12688/openreseurope.15581.2

**Published:** 2023-10-20

**Authors:** Jakub Bijak, Ariana Modirrousta-Galian, Philip A Higham, Toby Prike, Martin Hinsch, Sarah Nurse

**Affiliations:** 1 University of Southampton, Southampton, UK; 2 University of Western Australia, Perth, Australia; 3 University of Glasgow, Glasgow, UK

**Keywords:** Agent-based modelling, cognitive experiments, decision-making, interdisciplinary, migration, migration decisions, risk-taking, serious games

## Abstract

**Background:**

Agent-based modelling provides an appealing methodological choice for simulating human behaviour and decisions. The currently dominant approaches based on static transition rates or unverified assumptions are restrictive, and could be enhanced with insights from cognitive experiments on actual decision making. Here, one common concern is that standard surveys or experiments may lack ecological validity, limiting the extent to which research findings can be generalised to real-life settings. For complex, highly emotive decision-making scenarios, such as those related to migration, the typically used short, methodical survey questions may not appropriately map onto complex real-world decisions of interest. Immersive contexts may offer more accurate representations of reality, potentially enhancing the usefulness of experimental information in multi-disciplinary modelling endeavours.

**Methods:**

This pre-registered study of migration decisions, aimed at informing a multi-disciplinary construction of an agent-based model of migration, presents a choice-based interactive fiction game in which players make migration decisions to advance through a story. Participants ( *N* = 1000 Prolific users) were randomly assigned to one of four experimental conditions, three involving different renditions of the game attempting to create immersion, with the last condition presenting the decisions in standard survey format.

**Results:**

Although addressing the lack of ecological validity in survey data is important for improving agent-based modelling methodology, the experimental design used to tackle this issue, while responding directly to modelling needs, proved too complex. The created experimental conditions ended up too distinct from each other, involving stimuli that differed in quantity and content. This introduced several unintended and uncontrolled confounds, making it impossible to meaningfully interpret the results of this experiment on its own. Our results act as a cautionary tale for agent-based modellers, highlighting that the modelling needs should not override the principles of experimental design, and provide motivation for more rigorous research on this topic.

## Plain language summary

Agent-based models can be used to simulate people’s decisions and behaviour. To improve these models, information from surveys that ask people about their decisions and behaviours can be used. However, simple survey questions may not reflect real-life situations, so the answers someone gives in a survey may not match what they would do in real life. Games are more engaging and may offer more accurate representations of real-life situations. Therefore, they could be more useful than surveys. To examine this, we created a game in which players make migration decisions to advance through a story. We then conducted an experiment to investigate whether people that played this game made different migration decisions compared to people that took a survey. We did this to try to build an agent-based simulation of migration. Unfortunately, the experiment was too complicated, which made it difficult to clearly understand the results it produced. The way in which the game stories were set up could have mattered, too. We suggest carrying out more research on this topic, but with simpler experiments.

## Introduction

Agent-based modelling (e.g. Bonabeau, 2002), where simulated ‘agents’ represent people, groups, or institutions interacting with themselves and their environment, offer an appealing methodological choice for simulating human behaviour and gaining insights into the emergence of individual- and macro-level patterns. However, the currently dominant approaches, based on static transition rates or unverified assumptions for agents are limiting, and the existing secondary data are sometimes sparse for the task at hand. Under these circumstances, agent-based models could be enhanced with quantitative insights gained by collecting primary data on actual decision-making from, for example, surveys or cognitive psychology experiments ( Bijak *et al.*, 2021). By carrying out simulation experiments with models, and analysing their results, we can identify these model elements, such as parameters or rules, which would benefit the most from additional information. This information could have interdisciplinary provenance resulting from psychological or sociological insights.

At the same time, standard surveys and experiments may lack ecological validity. There has been a longstanding concern regarding the extent to which survey results about hypothetical decisions can be generalised to real-life settings ( Cicourel, 1982). For example, real-life migration decisions are often highly emotive, involve costly trade-offs, and may threaten one’s personal safety. These aspects are poorly represented by the short, methodical questions commonly used in surveys. With that in mind, it is reasonable to expect immersive contexts to offer more accurate and engaging representations of reality and provide better tools for capturing such complex decisions ( Rossetti & Hurtubia, 2020).

In light of this, the original research question of this study was the following: Does immersion affect risk-taking in the context of migration decisions? To address this query, in a preregistered exploratory study, we created a choice-based interactive fiction game in which participants made migration decisions to advance through a story. Choice-based interactive fiction games are fully text-based games where a player progresses by selecting from a list of possible actions ( Hausknecht *et al.*, 2020). Rafael (2018) suggested that emotional immersion, which denotes an emotional connection to the characters or social context of the game ( Zhang *et al.*, 2017), can be established in these types of games by gaining insight into the mental state of the characters. Relatedly, Lu *et al.* (2012) argued that creating positive affect for the protagonist, which can be achieved in various ways, such as making them an important source of information and beliefs, is vital for developing an immersive narrative. Consequently, we decided to use brief statements about the character’s internal thoughts and feelings, which we refer to as narrative, to foster immersion, since they provide insight into the mental state of the character and are believed to evoke emotion in readers, particularly empathy ( Keen, 2006).

Furthermore, McMahan (2003) argued that for a game to be immersive, the players’ actions must have a non-trivial impact on the game, which refers to a concept commonly known as player agency. However, Fendt *et al.* (2012) found that making players feel as if their actions had a non-trivial impact on the game, even when they did not, led to similar feelings of agency than actually making players’ actions have a non-trivial impact on the game. Therefore, considering that simulated agency is much easier to implement than actual agency, which would involve multiple story arcs and decision trees, we opted for the former. Specifically, created a sense of agency through repeated decision-making, with each decision being followed by a distinct piece of text outlining its consequence. These consequences made participants believe that their decisions were impactful; despite being dependent on the participants’ decisions, they did not affect any of the later decisions or the conclusion of the story.

In summary, to achieve immersion in our choice-based interactive fiction game, we (a) added narrative: brief statements about the character’s internal thoughts and feelings; and (b) created a sense of agency through repeated decision-making, with each decision being followed by a distinct piece of text outlining its consequence that, unbeknown to the player, did not have any impact on the game’s overall story or conclusion. Our game was designed on Qualtrics ( https://www.qualtrics.com/), a platform for creating and distributing online surveys. Despite not being game design software, Qualtrics allows for the implementation of HTML, CSS, and JavaScript, which are commonly used to create text-based games. For example, Twine ( https://twinery.org/), an online tool for creating choice-based interactive fiction games, uses these three programming languages. Connectedly, we decided to create a choice-based interactive fiction game because they require minimal programming compared to other types of games, which increases the likelihood that they will be used in research if they are shown to be better for capturing complex decision-making.

The design of our study was driven by the interdisciplinary needs of agent-based modelling endeavours, discussed in detail in Bijak *et al.* (2021). The process we followed for building an agent-based model of migration route formation was bottom up, starting from a simple representation of reality, and adding increasing detail in an iterative way. A statistical analysis carried out at each iteration of model building enabled identifying the key information gaps, which, if filled, could bring about the highest gains in terms of model performance. We attempted to fill these gaps where possible with existing secondary data, but also, specifically with respect to human decision making, with additional quantitative data from psychological experiments, such as those described in this paper, and several others ( Bijak *et al.*, 2021).

In this way, the primary data collected through experiments was intended to respond to specific modelling needs, in this case, related to modelling human decisions under varying levels of risk and reward, operationalised in the context of modelling migrant journeys through travel speed, perceptions of safety, and travel cost. Despite—or perhaps because of—close alignment to the modelling needs, dictated by the parameterisation of the model, the experiment we implemented eventually proved too complex, making the results on their own difficult to interpret. In particular, we identified trade-offs between conducting experiments that provide valuable psychological insights, and those that can inform agent-based models. Critically, to inform an agent-based model, large amounts of data for various different variables are required, which can only be obtained at the cost of experimental simplicity and thus control. In this paper, we present the lessons learned from this experimental exercise, and provide recommendations for future interdisciplinary research.

## Methods

### Participants

The study was carried out on Prolific ( https://www.prolific.co), with the participant pool approximately balanced in terms of sex and restricted to those fluent in English, residing in the UK at the time of the study, and with a Prolific approval rate above 90. After excluding three participants for completing the study in under 20% of the expected time and one for failing to complete the study, the final sample consisted of 1000 individuals. This included 491 female and 494 male participants, 10 individuals who identified as “other”, and five individuals who preferred not to disclose their gender. Participants were between the ages of 18 and 78 ( *M* = 37.07, *SD* = 13.29) and were paid at a rate of £5.00 per hour. The minimum age for our study was determined by Prolific, which requires participants to be at least 18 years old. We did not set a maximum age for our study.

To replicate this experiment without participant costs, we recommend advertising the study on social media websites such as Twitter and Reddit. This is an increasingly common data collection method for psychological research, which we plan on using for future studies.

### Experimental design and research variables

A between-subject, multi-group experimental design was used, with participants randomly assigned to one of four conditions: (a) narrative and agency; (b) narrative-only; (c) agency-only; and (d) survey (without narrative or agency). The independent variables were agency (yes, no), narrative (yes, no), decision stage (one, two, three), and trade-off (chance of success, safety, travel speed). The dependent variable was participants’ responses to migration decision(s). Ten control variables were collected for exploratory purposes, listed under ‘Demographic Questions’ below. All the authors and acknowledged team members tested the surveys prior to data collection, which led to the migration decisions being updated several times. Consequently, we pilot tested the final version of the study with 200 participants to identify potential problems, but we did not find any. 

### Migration decisions

 The experiment involved nine migration decisions, divided into three stages, each including three decisions. The three decisions in each stage corresponded to three different trade-offs, namely travel speed versus safety, chance of success versus safety, and chance of success versus travel speed. Although these different trade-offs were present at all three stages, the specific scenario and context for the decision differed between stages. For example, at Stage 1, the trade-off for travel speed versus safety involved either getting on an unsafe boat now or waiting for a safer boat; at Stage 2, the trade-off involved crossing a minefield or taking a long detour; and at Stage 3, waiting longer to cross with a safer smuggler versus crossing the border now with a less reputable one. This variability was implemented to enhance realism and ensure that the migration decisions seemed appropriately embedded within the current stage of the migration journey. However, as we discuss further in the conclusion, this strategy also led to difficulties in interpreting the results.

Participants were not presented with the same trade-off more than once. Therefore, at Stage 1, the first decision was randomly selected from the three trade-offs. At Stage 2, the second decision was randomly selected from the two decisions involving the remaining trade-offs. At Stage 3, the third decision presented the trade-off that had not been presented in either of the preceding stages (e.g., if a participant made a chance of success vs. travel speed decision at Stage 1 and a chance of success vs. safety decision at Stage 2, they would be presented with the safety vs. travel speed decision at Stage 3).

 The pools of potential migration decisions for the narrative-and-agency and narrative-only conditions were identical. However, in the narrative-only condition, participants were shown the outcome of the first two migration decisions (randomised in the survey) and only made the third migration decisions. In the agency-only condition, descriptions of the character’s thoughts and feelings were omitted. Finally, for the survey condition, the migration decisions were heavily stripped-down versions of those presented in the other conditions. All migration decisions and their different versions were created by the researchers following interdisciplinary discussions within the team about the requirements of the agent-based model of migration route formation ( Bijak *et al.*, 2021). To make the decisions as realistic as possible, we drew inspiration from both first-hand and second-hand accounts of migrant journeys. These were obtained from the website Telling the Real Story ( https://www.tellingtherealstory.org/en/) and from YouTube videos published by various news channels (e.g., BBC News and Sky news). The final conditions were set up so as to correspond as closely as possible to the simulated environment and decisions made by the agents in the model.

### Demographic questions

A total of 10 demographic and general questions were asked in all conditions, including questions about the participants’ emotional investment and sense of agency while playing (adapted from Jennett *et al.*, 2008), age, gender, number of children, how often they play games, whether they have ever considered migrating or have actually migrated, whether they think the number of immigrants in the UK should increase or decrease (adapted from The Migration Observatory, 2020), and how warm their feelings are towards migrants. Originally, we wanted to add these demographic variables as covariates in the analyses to determine how they influence risk-taking in the context of migration decisions. However, considering that the analyses already produced results that were impossible to meaningfully interpret, we decided not to add any more variables.

### Procedure

 No device restrictions were applied on Prolific. Before starting the study, participants were shown a combined information sheet and consent form (University of Southampton Ethics approval No. ERGO 68015). After reading the forms and providing informed consent, participants were asked to provide their Prolific IDs.

Participants were then randomly allocated to one of four conditions: (a) narrative and agency; (b) narrative-only; (c) agency-only; and (d) survey. Those in the narrative and agency condition made three migration decisions throughout a story that involved both narrative (i.e., descriptions of the character’s thoughts and feelings) and agency (i.e., repeated decision-making). Those in the narrative-only condition made one migration decision at the end of a story that involved narrative but no agency. Those in the agency-only condition made three migration decisions throughout a story that involved agency but limited narrative. Finally, those in the survey condition made three decisions presented as simple survey questions.

Once participants had finished their condition-specific task, they completed the demographic questions and were debriefed. The study’s length varied across conditions; the narrative and agency and the narrative-only conditions took approximately 15 minutes, the agency-only condition around 10 minutes, and the survey condition around 5 minutes. Since progression was entirely self-paced, the completion time varied between participants.

## Results

A series of logistic regression analyses were conducted to investigate the impact of condition, decision stage, and trade-off on participant’s decisions. Because participants in the narrative-only condition only made one decision (at the third stage), we first present a series of logistic regression analyses for the narrative and agency, agency-only, and survey conditions, including decision stage as a predictor (as well as condition and trade-off). These logistic regressions were conducted separately for each attribute of the decision (chance of success, safety, and travel speed). We then present logistic regressions on decisions made at the third stage only for all four conditions, excluding decision stage as a predictor (but including condition and trade-off). These logistic regressions are again conducted separately for each of the three attributes.

### Logistic regression analysis excluding the narrative-only condition

**Figure 1.**
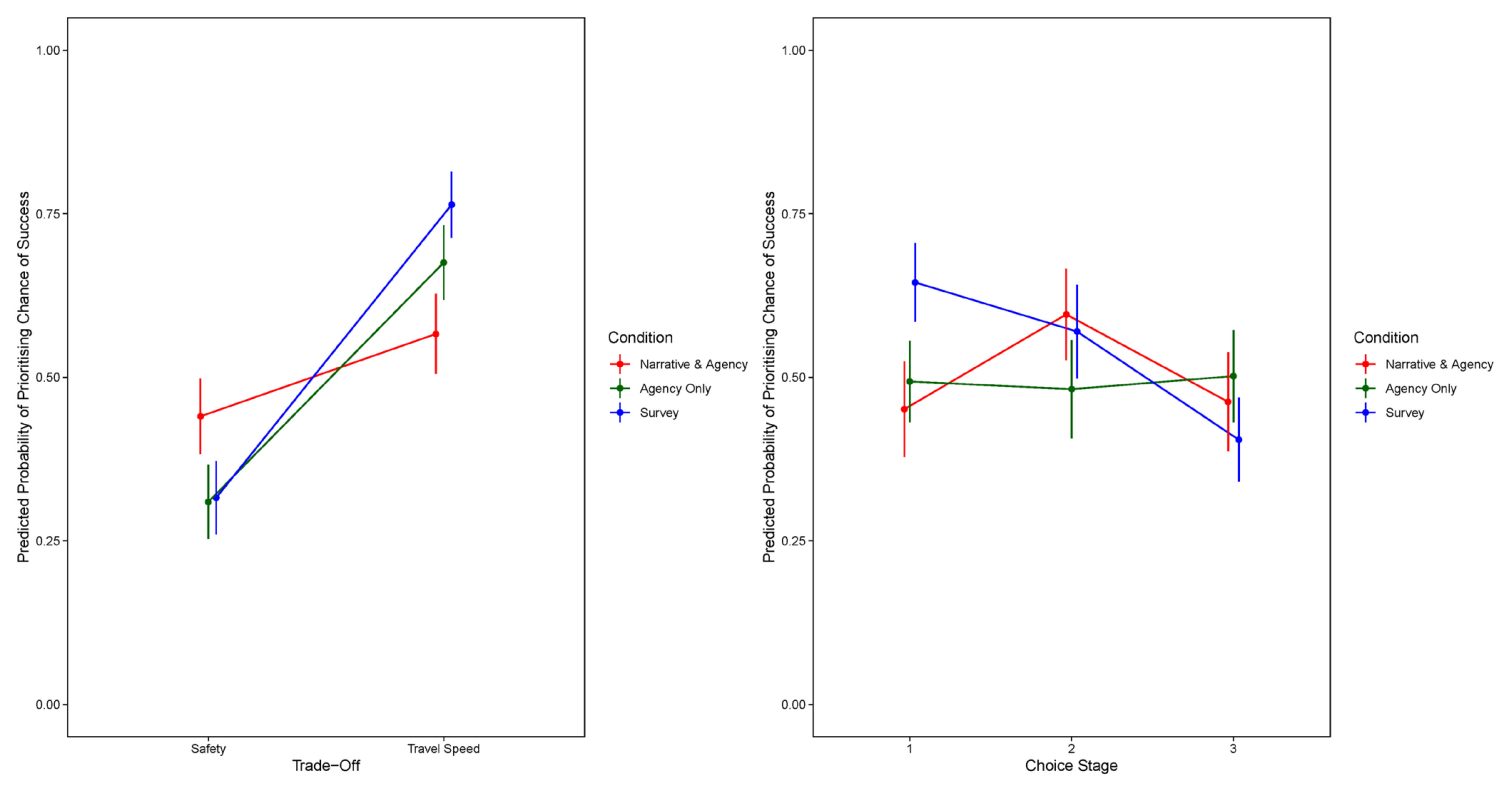
Predicted Probability of Prioritising Chance of Success, by Trade-Off and Condition (Top Panel) and by Decision Stage and Condition (Bottom Panel). The Narrative-Only Condition Is Excluded.

**Figure 2.**
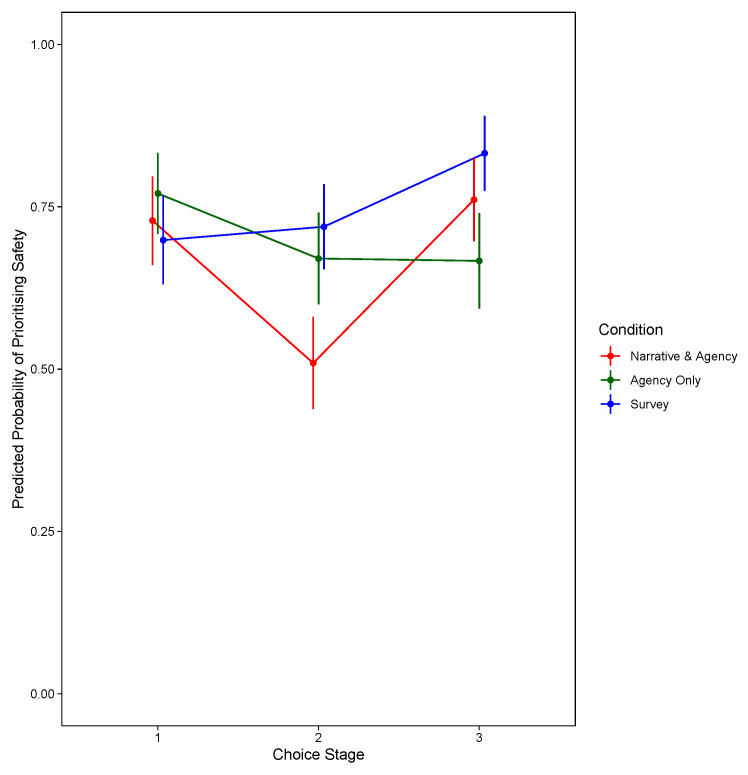
Predicted Probability of Prioritising Safety, by Decision Stage and Condition (Excluding Narrative-Only).

**Figure 3.**
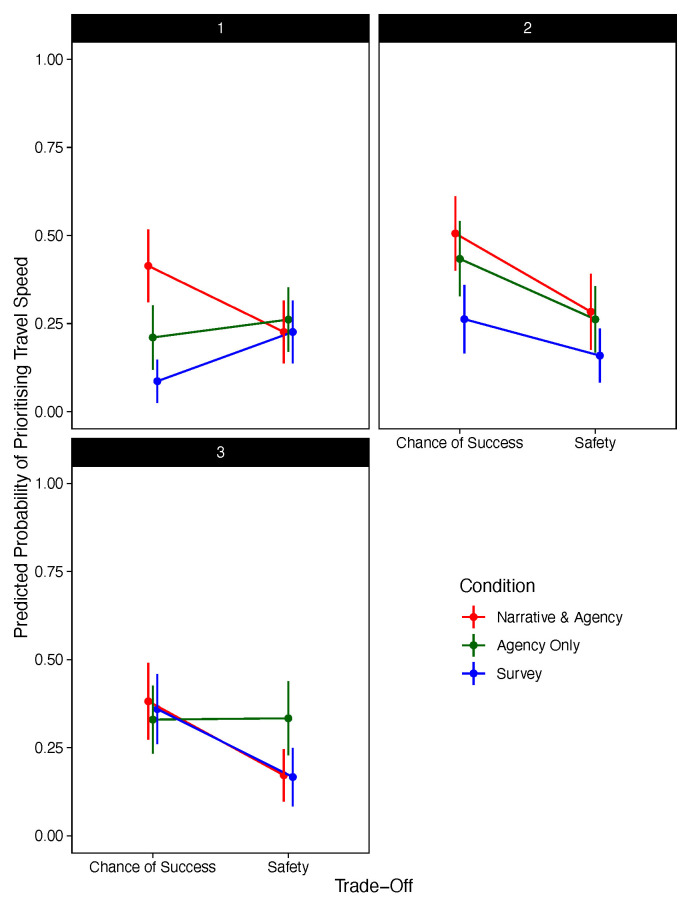
Predicted Probability of Prioritising Travel Speed Against Trade-Off, Decision Stage, and Condition (Excluding Narrative-Only).

A logistic regression analysis was conducted to investigate the relationship between the probability of prioritising one of the attributes of the decision (chance of success, safety, or travel speed) and the condition (narrative and agency, agency-only, or survey), decision stage (one, two, or three), and attribute that it was traded off against (chance of success, safety, or travel speed). Table 1 shows the analysis-of-deviance table produced by Type III Wald Chi-Squared tests. For the model prioritising chance of success, Figure 1 shows the significant two-way interaction between condition and trade-off and the two-way interaction between condition and choice stage. For the model prioritising safety, Figure 2 shows the significant two-way interaction between condition and choice stage, and for the model prioritising travel speed, Figure 3 presents the significant three-way interaction between condition, choice stage, and trade-off.

**Table 1.** Analysis of Deviance for Logistic Regression Models.

Factor(s)	*χ* ^2^	*df*	*p*
*Response Variable: Probability of Prioritising Chance* * of Success*			
Condition	7.00	2	.030
choice stage	36.27	2	< .001
trade-off	12.32	1	< .001
condition:choice stage	21.97	4	< .001
condition:trade-off	14.46	2	< .001
choice stage:trade-off	29.17	2	< .001
condition:choice stage:trade-off	7.92	4	.094
*Response Variable: Probability of Prioritising Safety*			
condition	7.00	2	.030
choice stage	36.27	2	< .001
trade-off	1.69	1	.194
condition:choice stage	21.97	4	< .001
condition:trade-off	5.11	2	.078
choice stage:trade-off	7.67	2	.022
condition:choice stage:trade-off	3.03	4	.553
*Response Variable: Probability of Prioritising Travel * *Speed*			
condition	26.04	2	< .001
choice stage	2.81	2	.246
trade-off	6.98	1	.008
condition:choice stage	14.86	4	.005
condition:trade off	13.85	2	< .001
choice stage:trade-off	0.19	2	.911
condition:choice stage:trade-off	10.84	4	.028

*Note*. The narrative-only condition was excluded.

### Logistic regression analysis excluding the first two decision stages

A further logistic regression analysis was conducted on third-stage decisions to investigate the relationship between the probability of prioritising one of the decision attributes (chance of success, safety, or travel speed) and the condition (all four conditions) and attributes it was traded off against (chance of success, safety, or travel speed). Table 2 shows the analysis-of-deviance table for models with response variables related to all three attributes of the decision. For the model prioritising chance of success, although the main effect of condition was significant, a post-hoc Tukey test showed no significant pairwise comparisons, with smallest *p* = .058. The main effect of trade-off was also significant, and a post hoc *z*-test showed that the probability of prioritising chance of success when it was traded off against safety ( *M* = .29, *SE* = .025) was significantly lower than the probability of prioritising chance of success when it was traded off against travel speed ( *M* = .66, *SE* = .026, *z* = –10.40, *p* < .001).

**Table 2.** Analysis of Deviance for Logistic Regression Models for Stage 3 Decisions only.

Factor(s)	*χ* ^2^	*df*	*p*
*Response variable: Probability of Prioritising Chance of* * Success*			
condition	8.39	3	.039
trade-off	15.01	1	< .001
condition:trade-off	3.71	3	.295
*Response variable: Probability of Prioritising Safety*			
condition	8.39	3	.039
trade-off	4.36	1	.037
condition:trade-off	3.32	3	.345
*Response variable: Probability of Prioritising Travel Speed*			
condition	1.84	3	.606
trade-off	9.75	1	.002
condition:trade-off	12.48	3	.006

*Note*. Decision stages one and two were excluded.

**Figure 4.**
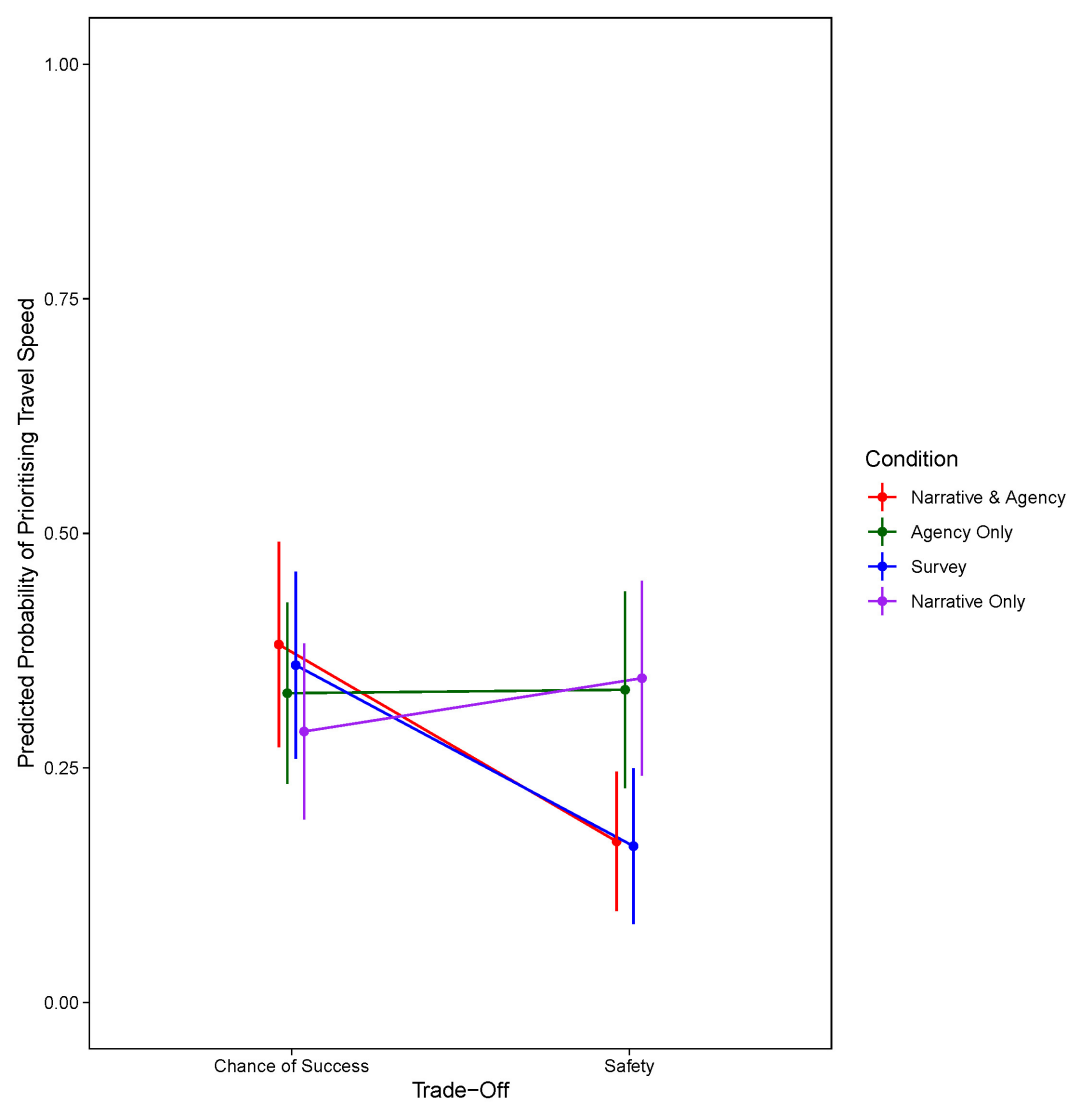
Predicted Probability of Prioritising Travel Speed Against Trade-Off and Condition at the Third Decision Stage.

For the model prioritising safety, the main effect of condition was again significant, and a post-hoc Tukey test showed that the probability of prioritising safety in the survey condition ( *M* = .83, *SE* = .030) was significantly higher than: (a) the probability of prioritising safety in the agency-only condition ( *M* = .67, *SE* = .037, *z* = –3.48, *p* = .003); and (b) the probability of prioritising safety in the narrative-only condition ( *M* = .66, *SE* = .038, *z* = 3.69, *p* = .001). Although the main effect of trade-off was also significant, the probability of prioritising safety when it was traded off against chance of success ( *M* = .71, *SE* = .025) was not significantly different from the probability of prioritising safety when it was traded off against travel speed ( *M* = .75, *SE* = .024, *z* = –0.97, *p* = .334). For the model prioritising travel speed, in addition to the summary statistics presented in Table 2, the significant two-way interaction between condition and trade-off are shown in Figure 4.

## Discussion and conclusion

 Interpreting the results of the experiment in a meaningful and generalisable way proved challenging. Firstly, both between and within conditions, participants experienced a different number of migration decisions (i.e., stimuli), some of which were presented in different contexts for the same attribute (e.g., boat crossing or landmines for safety), with different migration decisions presented in different orders. Therefore, although participants made conceptually analogous trade-offs, confounds made it difficult to meaningfully interpret the results. Furthermore, the conditions proved too distinct from each other, meaning that although the different renditions of each migration decision were intended to be analogous, there were unintended confounds. For example, participants could have made different migration decisions in the narrative and agency condition compared to the survey condition simply because in the former case, the text was substantially longer and contained more details than that in the latter.

 Overall, this study, even if exploratory in nature, illuminated some important tensions and trade-offs present in interdisciplinary research. On the one hand, there are important discipline-specific questions, in our case, around the lack of ecological validity in surveys. On the other hand, there is a need to align the experiment closely with the model needs through greater realism and participant immersion. Due to the need to satisfy the *interdisciplinary* modelling requirements, focusing specifically on quantitative information, from the point of view of experimental psychology, the resulting design ended up being too complex, and we could not obtain the *discipline-specific* psychological insights we had anticipated and desired. There are several significant differences and interactions, indicating that some signal may be coming through from the experimental data. However, because of issues with the study design, it is difficult to identify specific patterns and then interpret and attribute these findings to a particular variable, not to mention generalising them to different contexts.

Alternatively, idiosyncratic differences between the migration decisions across different conditions, as well as between versions of the same trade-off, designed to be analogous but differing in content and wording, dominated over everything else. Together with relatively small effects of immersion, this could have made the results inconclusive. In this interpretation, the main problem is the failure to anticipate the importance of the specific wording and the details of the situation in determining people's responses.

Ambiguous results notwithstanding, studying decision-making in richer and more ecologically valid conditions is a worthwhile endeavour, and the results of the ensuing experiments would enrich the toolkit of agent-based modellers, making the models more realistic and valid. However, as we tried to collect quantitative information about the decision processes, the approach reported in this paper proved at the same time too simple for the question at hand and for realistic applications in agent-based models, and too complex for proper formal analysis from the discipline-specific point of view of experimental psychology. This lesson suggests the need to follow a two-pronged approach, where the in-depth exploration of the topic of migrant decision is done by using qualitative methods, and the immersion effect as such can be only properly analysed with carefully designed—and simplified—experimental tools. 

In our case, the analysis of immersion is currently in progress, while the qualitative exploration was followed through a dedicated ethnographic study amongst Syrian and Afghan migrants, who were interviewed about their journeys to Europe ( Belabbas *et al.*, 2022). The results of this study enabled verifying or validating some of the key assumptions or results of modelling. Four aspects of decisions emerged as key: access to information, the extent of social capital (in particular, social networks), individual circumstances, and finally chance. This confirms the quantitative results reported in Bijak *et al.* (2021), where information and its sharing over networks were found to be some of the key drivers of model behaviour, while the high residual uncertainty of model outputs is corroborated by the salience of chance and unforeseen events for shaping migrant journeys.

We therefore hope that this paper acts not only as a cautionary tale, but also as motivation to conduct more rigorous and broader research on this topic, involving qualitative and quantitative aspects alike. Our main recommendation for future psychological research on immersion is to keep the design simple, with well-controlled differences between conditions carried out step-by-step, to allow for more interpretable results. For agent-based modelling of complex social processes, such as migration route formation, such results may be too simple, though, which suggests that the rules on which agent behaviour is modelled, should be based on qualitative information as well. The early results reported in Belabbas *et al.* (2022) are very promising in that regard. At the general level, the allure of interdisciplinary research needs to be moderated by discipline-specific rigour.

## Ethical approval

This study was approved by the University of Southampton, Ethics approval No. ERGO 68015. Participants provided informed consent to take part in this study and for their anonymised data to be published and uploaded to OSF (please see the combined participant information sheet and consent form at the beginning of all the Qualtrics surveys available on OSF: https://doi.org/10.17605/OSF.IO/PN9EW).

## Data Availability

Open Science Framework: Modirrousta-Galian, A. (2023). Investigating Immersion and Migration Decisions: A Cautionary Tale. *OSF*, DOI: https://doi.org/10.17605/OSF.IO/PN9EW, accessed on 31 January 2023. This project contains the following underlying data: Data (folder) Agency Only Condition_November 25, 2021_14.13.csv ( https://osf.io/8ewcf; raw data file for the agency-only condition) Narrative & Agency Condition_November 24, 2021_16.33.csv ( https://osf.io/uxp8d; raw data file for the narrative and agency condition) Narrative Only Condition_November 24, 2021_16.33.csv ( https://osf.io/279ux; raw data file for the narrative-only condition) Survey Condition_November 24, 2021_13.53.csv ( https://osf.io/uzea2; raw data file for the survey condition) Migration Decisions (folder) Migration Decisions.docx ( https://osf.io/gcmey; word document with all the migration decisions included in the experiment) Pilot Data (Folder) Agency Only Condition_November 18, 2021_19.35.csv ( https://osf.io/xdbjp; raw data file for the agency-only condition) Narrative & Agency Condition_November 19, 2021_15.04.csv ( https://osf.io/eup2c; raw data file for the narrative and agency condition) Narrative Only Condition_November 18, 2021_16.55.csv ( https://osf.io/rzdsv; raw data file for the narrative-only condition) Survey Condition_November 18, 2021_16.08.csv ( https://osf.io/8r9ac; raw data file for the survey condition Preregistration (folder) AsPredicted Preregistration.pdf ( https://osf.io/9uh2n; pdf file of the preregistration uploaded to the AsPredicted website) Qualtrics Surveys (folder) Agency_Only_Condition.docx ( https://osf.io/c8kgy; full Qualtrics survey for the agency-only condition) Narrative_Agency_Condition.docx ( https://osf.io/eqhkf; full Qualtrics survey for the narrative and agency condition) Narrative_Only_Condition.docx ( https://osf.io/frw7p; full Qualtrics survey for the narrative-only condition) Survey_Condition.docx ( https://osf.io/3j6kv; full Qualtrics survey for the survey condition) R Script (folder) chance of success analysis.R ( https://osf.io/rpncd; R script for the chance of success analysis) demographics.R ( https://osf.io/6wv8g; R script for the sample demographics) safety analysis.R ( https://osf.io/mkep2; R script for the safety analysis) travel speed analysis.R ( https://osf.io/kjt53; R script for the travel speed analysis) Data are available under the terms of the Creative Commons Attributions 4.0 International license (CC-By Attribution 4.0 International).
