# Supplementary material for: Investigating immersion and migration decisions for agent-based modelling: A cautionary tale
Source: Open Res Eur. 2024 Oct 29;3:34. Originally published 2023 Feb 20. [Version 3] doi: 10.12688/openreseurope.15581.3 (PMC11525092; doi:10.12688/openreseurope.15581.3)
Supplement: Peer review history

# Reviewer report: Reviewer response for version 3

**DOI:** 10.21956/openreseurope.20172.r45670

**Published:** 2025-01-23

**Authors:** Melania Borit

**Affiliations:** 1 Norwegian College of Fisheries Science, UiT The Arctic University of Norway, Tromsø, Norway

I would like to thank he authors for addressing my comments. I have no further comments.

If applicable, is the statistical analysis and its interpretation appropriate?

Yes

Are all the source data and materials underlying the results available?

Yes

Is the study design appropriate and is the work technically sound?

Partly

Is the work clearly and accurately presented and does it engage with the current literature?

Partly

Are the conclusions drawn adequately supported by the results?

Yes

Are sufficient details of methods and analysis provided to allow replication by others?

Partly

Reviewer Expertise:

Knowledge Integration / Methodologies for Interdisciplinary Research (including Agent-Based Modelling and Game Design)

I confirm that I have read this submission and believe that I have an appropriate level of expertise to confirm that it is of an acceptable scientific standard.

# Reviewer report: Reviewer response for version 3

**DOI:** 10.21956/openreseurope.20172.r45673

**Published:** 2024-11-08

**Authors:** Bruce Edmonds

**Affiliations:** 1 Centre for Policy Modelling, Manchester Metropolitan University, Manchester, UK

All my comments have been dealt with now. Happy for this to go ahead.

If applicable, is the statistical analysis and its interpretation appropriate?

Partly

Are all the source data and materials underlying the results available?

Yes

Is the study design appropriate and is the work technically sound?

Yes

Is the work clearly and accurately presented and does it engage with the current literature?

Yes

Are the conclusions drawn adequately supported by the results?

Partly

Are sufficient details of methods and analysis provided to allow replication by others?

Yes

Reviewer Expertise:

Agent-based policy modelling

I confirm that I have read this submission and believe that I have an appropriate level of expertise to confirm that it is of an acceptable scientific standard.

# Reviewer report: Reviewer response for version 3

**DOI:** 10.21956/openreseurope.20172.r45672

**Published:** 2024-10-30

**Authors:** Puyang Li

**Affiliations:** 1 Duke Kunshan University, Kunshan, Jiangsu, China

The authors have thoroughly and satisfactorily addressed my review comments from Version 2 in this revised Version 3. I approve indexing of this version of the manuscript.

If applicable, is the statistical analysis and its interpretation appropriate?

Yes

Are all the source data and materials underlying the results available?

Yes

Is the study design appropriate and is the work technically sound?

Yes

Is the work clearly and accurately presented and does it engage with the current literature?

Partly

Are the conclusions drawn adequately supported by the results?

Partly

Are sufficient details of methods and analysis provided to allow replication by others?

Partly

Reviewer Expertise:

geospatial modeling, GIS, remote sensing, agriculture modeling, smallholder management, China and Africa

I confirm that I have read this submission and believe that I have an appropriate level of expertise to confirm that it is of an acceptable scientific standard.

# Reviewer report: Reviewer response for version 3

**DOI:** 10.21956/openreseurope.20172.r45671

**Published:** 2024-10-30

**Authors:** Anne Goujon

**Affiliations:** 1 International Institute for Applied Systems Analysis, Laxenburg, Austria

Thank you to the authors for revising the manuscript according to the comments. The revisions have improved the readability and clarity of the exercise. I have no further comments.

If applicable, is the statistical analysis and its interpretation appropriate?

Not applicable

Are all the source data and materials underlying the results available?

Partly

Is the study design appropriate and is the work technically sound?

Yes

Is the work clearly and accurately presented and does it engage with the current literature?

Partly

Are the conclusions drawn adequately supported by the results?

Yes

Are sufficient details of methods and analysis provided to allow replication by others?

Partly

Reviewer Expertise:

Demography; ABM

I confirm that I have read this submission and believe that I have an appropriate level of expertise to confirm that it is of an acceptable scientific standard.

# Reviewer report: Reviewer response for version 2

**DOI:** 10.21956/openreseurope.18028.r41200

**Published:** 2024-07-04

**Authors:** Anne Goujon, Sebastian Poledna

**Affiliations:** 1 International Institute for Applied Systems Analysis, Laxenburg, Austria

Summary of the paper

 The paper titled "Investigating immersion and migration decisions for agent-based modelling: A cautionary tale" by Jakub Bijak et al. designed an experiment where participants made migration decisions within an interactive narrative game, aiming to gather more ecologically valid data than traditional surveys. The authors further aimed to use this interactive fiction game to enhance agent-based models (ABMs) of migration decision-making, which was left for future research. The authors further concluded that the complexity of the experimental design resulted in challenges in interpreting the findings, serving as a cautionary tale for interdisciplinary research.

The Idea to Use Choice-Based Interactive Fiction Games

 The idea of using a choice-based interactive fiction game to enhance ABMs is innovative and interesting. It is very plausible, that such games, that create a more immersive environment where participants can make decisions that mirror real-life scenarios, could provide richer and more accurate data on migration decision-making. Using this data on migration decision-making to inform ABMs could indeed enhance the results of modelling-based migration studies.

Data availability and preregistration

 It is very commendable that the authors made their data and code open-source and preregistered their study. This transparency and commitment to open science not only enhances the credibility of their work but also encourages collaboration and further advancements in the field. However, the link to the preregistration document which is listed in the data availability ( https://osf.io/9uh2n) does not work.

Presentation of the paper

 While the core idea is compelling, the presentation of the paper could benefit from some rewriting. It took us a while to understand the context of the study and what it is all about. It would be important to mention at one point, possibly in the introduction, that the experiment relates to migrants in an irregular situation. The reader understands this only when reading the Migration decisions paragraph. A short paragraph, mentioning the background for the experiment would be important and would ease the reading. At times, the structure and focus of the paper can make it challenging to follow the central theme. Some sections, including the introduction, seem to diverge from the main topic of the paper, which is the choice-based interactive fiction game. By focusing on the main contribution of the paper, the paper could be more accessible and engaging for readers.

Confusing claims about developing an ABM

 In addition to the above point, the present manuscript goes as far as creating the impression that it develops an ABM directly, which is not entirely accurate. For instance, phrases like "The process we followed for building an agent-based model of migration route formation was bottom up..." suggest direct ABM development. In reality, the focus is on experimental design and data collection to inform future ABMs. Clarifying this distinction would help readers better understand the paper's contributions and scope.

Conclusions of the paper

 The conclusion highlights the complexities and challenges faced in the study, noting that the experimental design ended up being too complex to yield clear results needed for agent-based modeling. While an agent-based modeler can understand this point and conclusion, it could also be made clearer for an interdisplinaryaudience.

Comment on the game design

 As the game design aspect is not within our area of expertise, we cannot provide a detailed critique. However, the use of interactive fiction games in this context seems a promising and innovative direction. It opens up new avenues for research and data collection that could significantly enhance the development of ABMs.

 In summary, while the paper presents an interesting idea to use interactive fiction games to inform ABMs, refining the presentation and focus could enhance its impact. The authors are encouraged to clearly differentiate between the central theme of the paper and the outlook for future research.

If applicable, is the statistical analysis and its interpretation appropriate?

Not applicable

Are all the source data and materials underlying the results available?

Partly

Is the study design appropriate and is the work technically sound?

Yes

Is the work clearly and accurately presented and does it engage with the current literature?

Partly

Are the conclusions drawn adequately supported by the results?

Yes

Are sufficient details of methods and analysis provided to allow replication by others?

Partly

Reviewer Expertise:

Demography; ABM

We confirm that we have read this submission and believe that we have an appropriate level of expertise to confirm that it is of an acceptable scientific standard, however we have significant reservations, as outlined above.

# Response

**Published:** 2024-09-26

**Authors:** Jakub Bijak

**Affiliations:** University of Southampton, UK

Thank you very much for your very useful comments and constructive suggestions - they have helped us improve the previous version, in particular by making the objectives and focus clearer. We respond to the main five points raised in your review below.

- Regarding the summary of the paper and the comments about open data, we thank you for your kind words and for making us aware of the broken link to the preregistration. We have now updated the link in question: https://osf.io/tpqxa.
- As regards the presentational side, and the need to make the central theme clearer, thank you for this important feedback. As other reviewers have picked up on this as well, we now try to better explain this dualism between the broader aims of assessing the feasibility of using experimental data in ABMs and the specific objectives of this study, related to immersion and risky migration decisions. We hope this comes through clearly now, both in the Introduction and Discussion sections. We have also added the recommended short explanatory paragraph (currently the third paragraph of the Introduction), with thanks for the suggestion.
- As to clarifying the claims about developing an ABM, in addition to making the objectives more transparent, as stated above, the text now reads: "In summary, in the background study to which our research was intended to contribute, the process of building an agent-based model of migration route formation was bottom-up, starting from a simple representation of reality, and adding increasing detail in an iterative way." We also hope the new additions to the text in the Introduction and elsewhere help alleviate any possible confusion.
- In addition, in the Discussion and Conclusions section, we have now added further explanations pertaining to agent-based modelling, which we hope will also speak to interdisciplinary readership.  
- Finally, in terms of the game design, thank you for the endorsement of the idea – and as for specific details, we have now added information about our game design, also in response to the comments of other reviewers, in a dedicated part of the Methods section.

# Reviewer report: Reviewer response for version 2

**DOI:** 10.21956/openreseurope.18028.r41201

**Published:** 2024-06-27

**Authors:** Puyang Li

**Affiliations:** 1 Duke Kunshan University, Kunshan, Jiangsu, China

This paper details an exploratory study that examined migrant decision-making at three stages of their journey using a game-based approach. Participants were assigned to one of four conditions, involving three different game renditions and one standard survey. While the complex design aimed to enhance the study of emotive decision-making beyond standard surveys, it ultimately resulted in data that were too difficult to interpret and integrate into agent-based modeling for complex social processes. Despite this, the research could be suitable for approval with significant revisions and elaborations.

 Firstly, I have concerns regarding the overall objective of this research. It appears the researchers aimed to create a game to enrich experimental insights as complements or additions to standard survey data, which could be fed into agent-based modeling to better reflect real-world conditions for complex social processes such as migration route formation. If this is accurate, then the research question proposed in the Introduction section is insufficient to summarize the overall objectives and provide a comprehensive overview of the paper.

 Secondly, I find it difficult to conclude that an agent-based model (ABM) can "only" be informed by simplified experimental information, although this is the central argument of the research. Therefore, I suggest cautiously proposing a simplified experimental procedure, as the results of this study only weakly support such inferences. This necessitates more elaboration in the discussion section on the potential reasons for the complexity/ambiguity of the experimental outcomes, i.e., could these stem from design issues or a lack of supportive data?

 Thirdly, I recommend adding a table describing the demographic questions and variables, detailing how they were formulated and identified. It is also essential to briefly note that if these variables were not considered as covariates, then how were they utilized to affect the experimental outcomes?

 Fourthly, there is no validation analysis in this paper. How did the researchers validate the experimental outcomes? I am unsure whether the ethnographic study of Syrian and Afghan migrants to Europe was intended for this purpose. Please clarify.

 Lastly, I have a minor question regarding the participants of the game. How were they selected? Were they migrants themselves, recreating their own migration experiences through the game? The identity of the participants could significantly affect the experimental outcomes.

If applicable, is the statistical analysis and its interpretation appropriate?

Yes

Are all the source data and materials underlying the results available?

Yes

Is the study design appropriate and is the work technically sound?

Yes

Is the work clearly and accurately presented and does it engage with the current literature?

Partly

Are the conclusions drawn adequately supported by the results?

Partly

Are sufficient details of methods and analysis provided to allow replication by others?

Partly

Reviewer Expertise:

geospatial modeling, GIS, remote sensing, agriculture modeling, smallholder management, China and Africa

I confirm that I have read this submission and believe that I have an appropriate level of expertise to confirm that it is of an acceptable scientific standard, however I have significant reservations, as outlined above.

# Response

**Published:** 2024-09-26

**Authors:** Jakub Bijak

**Affiliations:** University of Southampton, UK

Thank you very much for your very helpful comments and suggestions, in particular those related to clarifying the objectives clearer, which helped us focus the paper more clearly. We respond to the main five points raised in your review below.

- First, thank you for the request to clarify the objectives, which was very helpful. We have now added a clarification in the Introduction, to distinguish between the direct objectives of the experimental work (which we did not want to alter, given that this was the way in which they were preregistered), and the broader, indirect aims to test the feasibility of using the proposed approach for informing ABMs. We hope that, together with the changes to the conclusions, as discussed above, this distinction is clear and helps the reader navigate the paper.
- Second, regarding the point that an agent-based model (ABM) can "only" be informed by simplified experimental information, first of all, we apologise for the leftover "only" towards the end of the Introduction – indeed, this could have been misunderstood, and we have rephrased the sentence, which now reads: "Critically, to inform an agent-based model, large amounts of data for various different variables are required, with the likely trade-offs involving sacrificing experimental simplicity and control in order to obtain richer empirical material". We have also extended the discussion to include some of the suggestions above, for which thanks, and to signpost to the most recent results of the simplified experiment in the follow-up study (Modirrousta-Galian et al. 2024, https://doi.org/10.1177/10468781241242925).
- Third, following your recommendation, we have added a table describing the demographic questions and variables in the “Demographic and general questions” subsection of the Methods. We have left details of how they were formulated and identified in the text. Since these variables were not added as covariates in the analysis, due to the analysis already producing uninterpretable results, they were not utilised to affect the experimental outcomes. That is, we did not examine how these variables influenced migration-specific risk-taking. We have clarified this in the text.
- Fourth, related to the validation analysis, we apologise for the confusion in the previous version. Despite taking steps to ensure our migration decisions had a high level of content validity, we did not establish their construct validity. This is often done by examining convergent validity—the degree to which different measures designed to assess the same construct yield consistent results. However, to the best of our knowledge, no comparable measures of migration-specific risk-taking existed at the time of our study. We now discuss this in the "Migration decisions" subsection of the Methods.
- Fifth, regarding the recruitment of participants, all the selection criteria for participants are now provided in the "Participants" subsection of the Methods. We selected a participant pool that was approximately balanced in terms of sex, fluent in English, residing in the UK at the time of the study, and with a Prolific approval rate above 90. Migrant status was not one of the selection criteria. We did ask participants whether they ever considered migrating or actually migrated to a new country, but demographic characteristics were not included as covariates in the logistic regressions seeing as the analyses already produced results that were impossible to meaningfully interpret. Nonetheless, we completely agree that migrant status could significantly affect the experimental outcomes, which is why we investigated this in our aforementioned follow-up paper (Modirrousta-Galian et al. 2024).

# Reviewer report: Reviewer response for version 2

**DOI:** 10.21956/openreseurope.18028.r35755

**Published:** 2024-01-02

**Authors:** Melania Borit

**Affiliations:** 1 Norwegian College of Fisheries Science, UiT The Arctic University of Norway, Tromsø, Norway

Dear authors,

 Thank you for revising your submission.

 I read your answer to my comments and here is my response:

  

1. The text added in the introduction section regarding your choices vis-à-vis immersion should be moved in the methods section.
2. In the introduction and also in the methods section, make clear what is the relation between experiments and playing the game.
3. This comment is related to my original 5 ^th^ comment (which was misinterpreted; sorry for that, I should have formulated it in a clearer way). The method section has to include a clear and concrete description of how the game was designed, using terminology from the respective field. There is plenty of research on game design and even more on game analysis. Your own statement “At the general level, the allure of interdisciplinary research needs to be moderated by discipline-specific rigour” applies here, too, as game design is a discipline in itself.
4. Furthermore, most likely your game is not the first one of this kind. Any thoughts about this? Have you checked for such games? Have you found any?

I am looking forward to these details related to the design of the game!

If applicable, is the statistical analysis and its interpretation appropriate?

Yes

Are all the source data and materials underlying the results available?

Yes

Is the study design appropriate and is the work technically sound?

Partly

Is the work clearly and accurately presented and does it engage with the current literature?

Partly

Are the conclusions drawn adequately supported by the results?

Yes

Are sufficient details of methods and analysis provided to allow replication by others?

Partly

Reviewer Expertise:

Knowledge Integration / Methodologies for Interdisciplinary Research (including Agent-Based Modelling and Game Design)

I confirm that I have read this submission and believe that I have an appropriate level of expertise to confirm that it is of an acceptable scientific standard, however I have significant reservations, as outlined above.

# Response

**Published:** 2024-09-26

**Authors:** Jakub Bijak

**Affiliations:** University of Southampton, UK

Thank you very much for your very helpful comments and clarifications - they have certainly helped us sharpen the argument and improve the paper. We respond to the main four points raised in your review below.

- First, we have moved most of the text regarding immersion from the Introduction to a new subsection in the Methods titled "Game Design" (see also the third comment below). However, we have kept a description of the two elements incorporated in the game to foster immersion in the Introduction, since they are important for making the relation between the experiment and playing the game clear, also referring to your second comment.
- Second, we have clarified in the Introduction and the Methods that the experiment involved four conditions, each of which had participants play what is essentially a different version of the game.
- Third, we apologise for misinterpreting your previous comment from the first review. We have now added a subsection in the Methods titled "Game Design" that outlines the rationale behind the key design choices for our game.
- Fourth, thank you for raising the important consideration of novelty. To the best of our knowledge, we are the first to create a text-based game specifically to examine migration-specific risk-taking. That being said, there are other text-based games about migration more generally (e.g., BBC's "Syrian Journey" or IOM's "Migrant Journey"), as well as similar story-based games on popular gaming platforms, such as Steam, which include, for example, "Bury Me, My Love", or "The Night Fisherman"). Some of these games have been used in empirical studies to examine their impact on certain outcomes (e.g., prejudice; Lee & Chen, 2023). However, as far as we know, these games are not specifically designed for academic research, so they have more complex designs that do not facilitate the investigation of players' in-game actions. Therefore, these games differ considerably from ours in terms of research applicability, focus, and design. We have now included these comments in the Introduction of the paper.

# Reviewer report: Reviewer response for version 1

**DOI:** 10.21956/openreseurope.16841.r30877

**Published:** 2023-05-16

**Authors:** Melania Borit

**Affiliations:** 1 Norwegian College of Fisheries Science, UiT The Arctic University of Norway, Tromsø, Norway

This paper presents an exploratory study that was carried out to test an experimental design more suitable to developing complex, highly emotive decision-making scenarios than standard surveys. The created experimental conditions ended up to be too complicated and made it impossible to interpret the results of this experiment.

 As expressed by the authors themselves, the value of this paper lies in being a story from which other researchers can learn (“cautionary tale”). However, since one could have hypothesized already from the beginning of the study that the results would be ambiguous and difficult to interpret because of the complexity of the experimental design, it is important to understand why the study was carried out in this form (how were the design decisions taken, which decisions are related to working in an interdisciplinary team and which decisions are connected more to the discipline-specific thinking, who were the members of the team and what was their role etc.). As such, the study would be useful from a methodological point of view for interdisciplinary teams working with agent-based modelling. Thus, I suggest enriching the description of the methodology. From my perspective, at least the following aspects have to be described in more details:

- What exactly is the research question / objective / scope of this paper? The authors have to be clearer about that, in order to help the reader through its journey of understanding the cautionary tale.
- How did the team work with the topics of immersion, narrative, and agency? Did the team have expertise in game design? Did the team review the game design research field in order to come up with their design choices related to these topics? There are a multitude of previous studies about the use of narrative as a method to create immersion and about the use of agency for the same purpose; it is not clear whether and to what extent the team working with the current study made use of this previous research.
- How was the narrative on migration designed? The reference (Bijak et al., 2021) is a whole book, which makes it difficult for the reader of the current study to trace back the decisions of the team in order to learn about the interplay between interdisciplinarity and discipline-specific thinking.
- What was the basis for formulating the demographic questions?
- How was the gameplay designed?
- Though the data collection was intended to be used for agent-based modelling endeavours, the current paper does not report anything related to the actual work with this model, thus it is not clear how the experiment is connected to agent-based modelling. More explanations are needed regarding how, theoretically speaking, the results of this study would be connected to agent-based modelling and how the agent-based modelling team worked with designing the experiment so that it fits their modelling needs (said to be interdisciplinary). If such explanations are not added, then the mentions to agent-based modelling have to be down-played (otherwise, the focus on the agent-based model (as expressed in the title, abstract, and introduction) might be interpreted as misleading).

As a small comment, I would rename Conclusion into Discussion and Conclusion.

If applicable, is the statistical analysis and its interpretation appropriate?

Yes

Are all the source data and materials underlying the results available?

Yes

Is the study design appropriate and is the work technically sound?

Partly

Is the work clearly and accurately presented and does it engage with the current literature?

Partly

Are the conclusions drawn adequately supported by the results?

Yes

Are sufficient details of methods and analysis provided to allow replication by others?

Partly

Reviewer Expertise:

Knowledge Integration / Methodologies for Interdisciplinary Research (including Agent-Based Modelling and Game Design)

I confirm that I have read this submission and believe that I have an appropriate level of expertise to confirm that it is of an acceptable scientific standard, however I have significant reservations, as outlined above.

# Response

**Published:** 2023-10-02

**Authors:** Jakub Bijak

**Affiliations:** University of Southampton, UK

Thank you very much for all excellent comments, which are gratefully received. We have now attempted clarifying our methodological and design decisions in greater detail, as set forth below.

- *What exactly is the research question / objective / scope of this paper? The authors have to be clearer about that, in order to help the reader through its journey of understanding the cautionary tale.*

We have included our preregistered research question in the extended version of the Introduction.

- *How did the team work with the topics of immersion, narrative, and agency? Did the team have expertise in game design? Did the team review the game design research field in order to come up with their design choices related to these topics? There are a multitude of previous studies about the use of narrative as a method to create immersion and about the use of agency for the same purpose; it is not clear whether and to what extent the team working with the current study made use of this previous research.*

We have now reviewed the literature on immersion, narrative, and agency to develop the design of our game. We have added more information about this in the Introduction.

- *How was the narrative on migration designed? The reference (Bijak et al., 2021) is a whole book, which makes it difficult for the reader of the current study to trace back the decisions of the team in order to learn about the interplay between interdisciplinarity and discipline-specific thinking.*

We have included this information in the Methods section, after the reference. In essence, the design was iterative: the analysis of successive versions of the migration model being developed pointed to gaps in the knowledge about the behaviour of agents. We then attempted filling some of these gaps with knowledge from psychological experiments, of which this is one.

- *What was the basis for formulating the demographic questions?*

We have added information about this in the Methods section.

- *How was the gameplay designed?*

There are two aspects to this question, related to: (1) details on what software we used to design the gameplay; and (2) details on the design of the gameplay itself. In the revised version, we aim to address both. Regarding (1), we have added information about this in the Introduction. Regarding (2), gameplay (how a game is played) is directly related to a game’s genre (a category of games with similar gameplay). Therefore, our decision to create a choice-based interactive fiction game determined the gameplay, which is described throughout the manuscript. Nevertheless, we have added information about why we decided to create a choice-based interactive fiction game rather than any other type of game, which was previously missing from the manuscript, to the Introduction.

- *Though the data collection was intended to be used for agent-based modelling endeavours, the current paper does not report anything related to the actual work with this model, thus it is not clear how the experiment is connected to agent-based modelling. More explanations are needed regarding how, theoretically speaking, the results of this study would be connected to agent-based modelling and how the agent-based modelling team worked with designing the experiment so that it fits their modelling needs (said to be interdisciplinary). If such explanations are not added, then the mentions to agent-based modelling have to be down-played (otherwise, the focus on the agent-based model (as expressed in the title, abstract, and introduction) might be interpreted as misleading).*

Thank you very much for these really important points. We now signalled these links at the very beginning of the paper (why the information was needed), and made them more explicit in the expanded discussion in the later part of the Introduction. *As a small comment, I would rename Conclusion into Discussion and Conclusion.* We have renamed the final section as suggested.

# Reviewer report: Reviewer response for version 1

**DOI:** 10.21956/openreseurope.16841.r30875

**Published:** 2023-05-16

**Authors:** Bruce Edmonds

**Affiliations:** 1 Centre for Policy Modelling, Manchester Metropolitan University, Manchester, UK

A game is used to investigate possible decisions a migrant might make at 3 stages of their simulated journey. The researchers gathered some data about the participants and then secretly assigned them to one of four conditions: agency or not x narrative or not (where agency means repeated decision-making and narrative means they are supplied with a narrative of the migrants feelings, motivations etc.). This data was analysed using standard stats. The basic research is interesting enough to be published in ORE.

 Their conclusion was that, even given this relatively simple data that it was too complex to be translated into a simulation and hence a simpler experiment was needed. This is, to me, an astounding conclusion. That people's decision-making in such circumstances is complex reflects reality - seeking to force reality to be even simpler to fit one's approach is unscientific. The conclusion I would come to is that the framework that the researchers had in mind for modelling the decision-making is inadequate and their data collection too restricted.

 Rather, I suggest that the researchers should collect more informative data about how the subjects come to their decisions using interviews or narrative questions. This may reveal (a) the menu of strategies that the agents are using and (b) some of the context-dependency involved in picking strategies. Agent-based modelling is well-suited to simulating such context-dependent strategies. The problem lies in the bias of the researchers to use experimentation rather than the simulation stage to try and understand what is happening. Narrative data could give valuable clues as to what is happening here. Rather than simplify the design (which is already very simple compared to reality) they should broaden their data collection and utilise the full power of agent-based modelling.

 Once the range of strategies and contexts are discovered the appropriate analysis of the single valued data should be more obvious. How people decide is complex - one either faces this or accepts one is not modelling real decision-making (so any simulation that results would be no guide to observed migration behaviour).

 I suggest that the researchers expand their conclusions with consideration of a broader range of interpretations and future directions for their research (even if they ultimately decide to follow their stated path).

If applicable, is the statistical analysis and its interpretation appropriate?

Partly

Are all the source data and materials underlying the results available?

Yes

Is the study design appropriate and is the work technically sound?

Yes

Is the work clearly and accurately presented and does it engage with the current literature?

Yes

Are the conclusions drawn adequately supported by the results?

Partly

Are sufficient details of methods and analysis provided to allow replication by others?

Yes

Reviewer Expertise:

Agent-based modelling, collecting data from experiments and observations

I confirm that I have read this submission and believe that I have an appropriate level of expertise to confirm that it is of an acceptable scientific standard, however I have significant reservations, as outlined above.

# Response

**Published:** 2023-10-02

**Authors:** Jakub Bijak

**Affiliations:** University of Southampton, UK

Thank you very much for these comments - we found them really insightful. We have tried incorporating them throughout the new version of the paper, changing the overall message and making it more nuanced (please see especially the new discussion in the Discussion and Conclusion section). The gist of the current argument is that, as we tried to collect quantitative information about decision processes, the approach we tried to follow was at the same time too simple for the question at hand (and for ABM applications more broadly), and too complex for carrying out a proper formal analysis from the discipline-specific point of view (experimental psychology). The answer we now propose is a two-pronged approach, where the in-depth exploration of the migration decisions has been carried out by using qualitative methods, which we indeed followed in Belabbas et al. (2022), while the immersion effect as such can be only properly analysed with carefully designed and analysed experimental tools (which is still work in progress).
